# Phylogeography in *Galaxias maculatus* (Jenyns, 1848) along Two Biogeographical Provinces in the Chilean Coast

**DOI:** 10.1371/journal.pone.0131289

**Published:** 2015-07-10

**Authors:** Claudio A. González-Wevar, Pilar Salinas, Mathias Hüne, Nicolás I. Segovia, Luis Vargas-Chacoff, Marcela Astorga, Juan I. Cañete, Elie Poulin

**Affiliations:** 1 GAIA-Antártica, Universidad de Magallanes, Punta Arenas, Chile; 2 Laboratorio de Ecología Molecular, Instituto de Ecología y Biodiversidad, Departamento de Ciencias Ecológicas, Facultad de Ciencias, Universidad de Chile, Ñuñoa, Santiago, Chile; 3 Fundación Ictiológica, Providencia, Santiago, Chile; 4 Instituto de Ciencias Marinas y Limnológicas, Facultad de Ciencias, Universidad Austral de Chile, Valdivia, Chile; 5 Instituto de Acuicultura, Universidad Austral de Chile CIEN Austral Puerto Montt, Puerto Montt, Chile; 6 Departamento de Recursos Naturales, Universidad de Magallanes, Punta Arenas, Chile; Chinese Academy of Sciences, CHINA

## Abstract

Major geologic and climatic changes during the Quaternary exerted a major role in shaping past and contemporary distribution of genetic diversity and structure of aquatic organisms in southern South America. In fact, the northern glacial limit along the Pacific coast, an area of major environmental changes in terms of topography, currents, and water salinity, represents a major biogeographic transition for marine and freshwater species. We used mitochondrial DNA sequences (D-loop) to investigate the consequences of Quaternary glacial cycles over the pattern of genetic diversity and structure of *G*. *maculatus* (Pisces: Galaxiidae) along two biogeographical provinces in the Chilean coast. Extreme levels of genetic diversity and strong phylogeographic structure characterize the species suggesting a low amount of influence of the last glacial cycle over its demography. However, we recognized contrasting patterns of genetic diversity and structure between main biogeographical areas here analyzed. Along the Intermediate Area (38°–41° S) each estuarine population constitutes a different unit. In contrast, Magellanic populations (43°–53° S) exhibited low levels of genetic differentiation. Contrasting patterns of genetic diversity and structure recorded in the species between the analyzed biogeographic areas are consistent with the marked differences in abiotic factors (i.e., different coastal configurations, Quaternary glacial histories, and oceanographic regimes) and to inherent characteristics of the species (i.e., salt-tolerance, physiology, and reproductive behavior).

## Introduction

Quaternary glaciations, and especially the Last Glacial Maximum (LGM) 25–18 Ka, caused major shifts in the distribution of the biota, particularly at higher latitudes [1–3]. Until the last two decades, phylogeographic studies in higher latitude taxa were highly biased to Northern Hemisphere taxa and most of them were focused on terrestrial organisms [4,5]. However, during the last decade, more and more genetic data has been accumulated showing the role of the Quaternary glaciations in the southern tip of South America. Such studies include population-based and phylogeographic analyses in different Patagonian taxa from marine invertebrates [6–8] to mammals [9–11]. The glacial history of South America is generally well understood; ice sheet advances and retreats during the Quaternary generated major shifts in sea level, climate and landscape [12–15]. During the LGM the Patagonian Ice Sheet (∼ 480,000 km^2^) expanding from 35°S to 56°S covered most of the Pacific Magellanic fjords and channels with an ice-volume of more than 500,000 km^3^ [12,16–17]. Hence, the geomorphology of Patagonia during the last 2 million years has not always remained stable and major physical changes during Quaternary glacial periods markedly affected current patterns of genetic diversity and structure in aquatic organisms, particularly in freshwater and marine near-shore ecosystems [18,19]. In fact, radical glacial landscape changes resulted in periodic extinction of fauna associated to these ecosystems allowing the colonization of vacant niches, as well as creating opportunities for geographical isolation and speciation [7,20,21].

According to Zemlak [22], postglacial patterns of dispersal in southern South American fishes includes the presence of several independent Quaternary glacial refugia on the east side of the Andes, along the Patagonian Steppe [23–25]. Along the west slope of the Andes refugia could have been located both within [9,25–27] and outside of the glacier limits [24–26,28]. Absence of genetic structure and strong signal of recent demographic growth support the hypothesis of rapid postglacial expansion in shallow Magellanic marine benthic invertebrates [6,8]. Finally, there is evidence that some groups of marine organisms re-colonized the southern tip of South America from geographically distant ice-free regions [29,30].

The Family Galaxiidae includes ~ 50 species of Gondwanan-distributed fishes commonly found in cool-temperate regions in the Southern Hemisphere. All species inhabit freshwater ecosystems, although, some of them present a migratory life stage with a salt-tolerant larval phase known as whitebait. These diadromous species exhibit considerably greater dispersal capabilities than those that are non-migratory [19]. Among diadromous species, *Galaxias maculatus* is one of the most widely distributed freshwater fish in the planet and exhibits a Gondwanic distribution. Populations of *G*. *maculatus* are currently found in freshwater and marine ecosystems of southern Australia, Tasmania, New Zealand and surrounding islands, and in the temperate latitudes of South America and the Falkland/Malvinas Islands [31]. The transoceanic distribution of *G*. *maculatus* has been explained by vicariance [32] and dispersal [33] hypotheses, but molecular evidence suggests that populations dispersed from Australia to other locations following the West Wind Drift [34–36]. Along the Chilean coast, *G*. *maculatus* includes landlocked and migratory populations in freshwater, estuarine and marine ecosystems [37] in two of the three Chilean biogeographic areas, namely the Intermediate Area (30°S–42°S) and the Magellanic Province (42°S–56°S) [38]. Phylogeographic analyses in *G*. *maculatus* detected a small influence of the last glacial cycles over the genetic diversity of the species in South America. In fact, female populations size in *G*. *maculatus* seems to have remained relatively constant until ~ 500 Ka when populations sizes increased exponentially reaching contemporary levels [22]. More recently, Zemlak et al. [39] recognized that *G*. *maculatus* experienced a severe genetic bottleneck between 1.1 Ma and 0.6 Ma, a period when the Patagonian Ice Sheet reached its maximum northward expansion [13,15]. Following this, the species experienced a strong population recovery during the late Quaternary (400 Ka) probably associated to long and warm interglacial periods [39].

The Chilean coast represents an interesting area to evaluate the relative effect of glaciological history, oceanography, and habitat discontinuities for aquatic organisms. The presence of extensive ice sheets during the glacial periods of the Quaternary likely eradicated recurrently most of the suitable freshwater and brackish marine habitats along the Magellanic Province while the Intermediate Area was considerably less impacted. In this context, *Galaxias maculatus* constitutes a suitable model to infer how historical and contemporary climatic processes have shaped the distribution of the genetic diversity in the species. Here we evaluated the consequences of the Quaternary glacial cycles over the pattern of genetic diversity and structure in a diadromous species with special emphasis in coastal and estuarine populations along two biogeographic marine areas that were differentially affected by ice advances and retreats.

## Materials and Methods

### Ethics Statement

This work was conducted using puye (*Galaxias maculatus*) as model study, a common freshwater and brackish fish species along the Chilean coast. The species is not protected and is considered as vulnerable in Central Chile and as out of danger in the Magellanic Province. Permission to undertake field works and collect specimens was issued by the Chilean Fisheries Service Director (Pablo Galilea Carrillo), under the technical memorandum (427/2011). The Instituto de Ecología y Biodiversidad (IEB/12-2011) and Chilean Fisheries Service (SERNAPESCA 427/2011) ethic committees approved sampling protocols and experiments. For this, we compiled with local legislation and the Convention on Biological Diversity.

### Sample collection, DNA extraction, PCR amplification and sequencing

**Fig 1:**
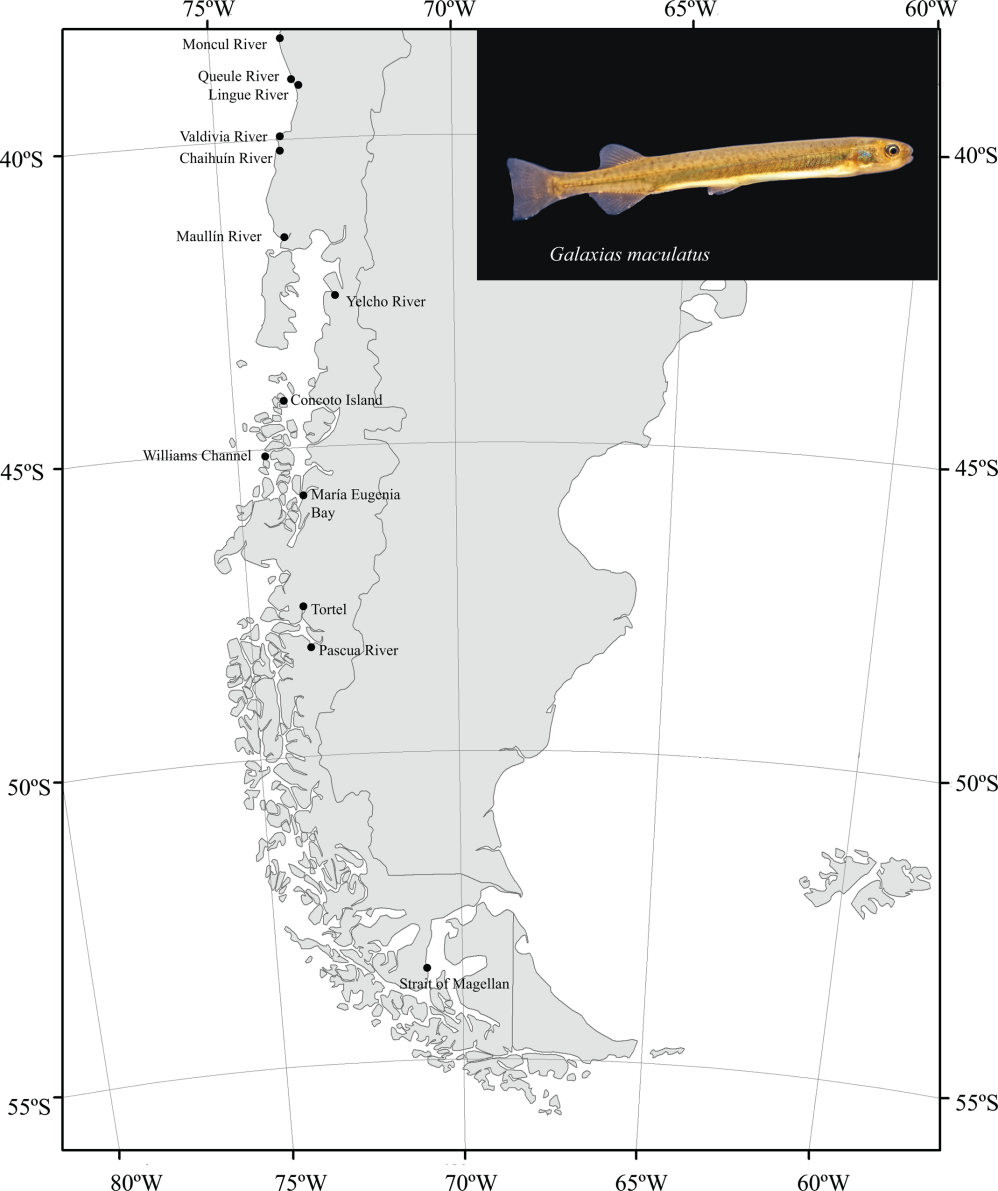
Sampling localities of *Galaxias maculatus* along the Chilean coast.

Individuals were collected between 2007–2014 in 13 estuarine localities along two main biogeographic areas in the Chilean Coast. Firstly, we included six localities from the Intermediate Area, between 38°S and 42°S, outside the direct influence of the LGM Patagonian Ice Sheet: Intermediate Area localities were: 1) Moncul River (38°42’S; 73°24’W); 2) Queule River (39°17’°S; 73°10’W); 3) Lingue River (39°26’S; 73°09’W); 4) Valdivia River (39°53’S; 73°24’W); 5) Chaihuín River (40°01’S; 73°27’W), and 6) Maullín River (41°35’S; 73°38’W). Secondly, we included seven localities from the Magellanic Province, between 43° S and 53° S, within the influence of the LGM Patagonian Ice Sheet. Magellanic localities were: 1) Yelcho River (43°03’S; 72°33’W), 2) Concoto Island (44°06’S; 73°43’W); 3) Williams Channel, Rivero Island (45°25’S; 74°21’W); 4) María Eugenia Bay (45°55’S; 73°31’W); 5) Tortel, Calén Fjord (47°45’S; 73°32’W); 6) Pascua River, Calén Fjord (48°15’S; 73°18’W); and 7) Strait of Magellan (53°14’S; 70°59’W; Fig 1).

Whole specimens were fixed in ethanol (95%) and DNA was prepared from muscle tissue using a salting-out method [40]. A partial fragment of the mitochondrial Control Region (D-loop) was amplified using specific primers GAL-F 5’–TAA CTC TCA TTA ACT AAA G– 3’ and GAL-R 5’–TGA TAG TAA AGT CAG CAA GCC– 3’) designed from the complete mitochondrial genome of the species (ACN: AP004104) [41]. PCR amplifications were performed in a 25 μl reaction containing 2.5 μl 10X Buffer (50 mM KCl, 10 mM Tris-HCl, pH 8.0), 1.0 μl of 50 mM MgCl_2_, 200 mM dNTPs, 0.5 μl of each primer (10 pg/μl), 1 U Taq (Invitrogen), 17.5 μl of double-distilled water and 5 ng of DNA. Thermal cycling parameters included an initial denaturation step at 94°C for 5 min, followed by 35 cycles at 94°C for 90 sec, 60.7°C for 90 sec, and 72°C for 90 sec, and a final 10 min extension at 72°C. PCR amplification products were purified using QIAquick Gel Extraction Kit (QIAGEN) and sequenced in both directions with an Automatic Sequencer ABI3730 x 1 at Macrogen Inc. (Seoul, Korea).

### Genetic diversity and population structure in *Galaxias maculatus*

D-Loop sequences were edited using Proseq v. 3.5 [42] and aligned with ClustalW [43]. New D-loop haplotypes recorded in the species were deposited in GenBank under the Accession Numbers KP298433—KP298673 and KP336557—KP336665. We performed a DNA saturation analysis following Xia et al. [44] to evaluate how saturation of transitions is accumulated in relation to nucleotide divergence in the whole data set. Levels of genetic polymorphism were estimated using standard diversity indices including: the number of haplotypes (*k*), the number of segregating sites (*S*), haplotypic diversity (*H*), the average number of pairwise differences (*П*), and nucleotide diversity (*π*) for each locality, for each main area, and for the whole D-loop data set using DnaSP v.5.00.07 [45]. We performed neutrality statistical tests (Tajima’s D and Fu’s F_S_) for each locality, area, and for the whole data set to estimate whether D-loop sequences in *G*. *maculatus* deviate from expectations under a neutral model. Considering the high levels of genetic diversity previously estimated in the species [22,39,41] we determined the levels of genetic differentiation between the analyzed localities using mean pairwise differences (N_ST_) following Pons and Petit [46] in Arlequin v. 3.5 [47]. The statistical significance of genetic differences between localities was estimated using permutation tests using 20,000 iterations of haplotype identities. Similarly, we estimated the levels of genetic differentiation of subpopulations using the nearest-neighbor statistic (S_nn_) that measure how often nearest neighbors (in sequence space) of sequences are from the same locality in geographic space [48]. The statistical significance of S_nn_ analyses was determined through a permutation test using 10,000 iterations. We inferred the spatial genetic structure in the species by estimating the number and composition of groups that were most differentiated based on sequence data with an Analysis of Molecular Variance (AMOVA) following Excoffier et al. [47] in Arlequin. AMOVA is a popular method that uses multiple spatial scales in statistical methods to characterize spatial genetic structure by partitioning it into: within populations, among populations within groups and among groups. Finally, we performed a test for isolation by distance using a Mantel test with 1,000 permutations in Arlequin to determine the correlation between Slatkin’s linearized localities genetic differentiation [49] and the linear geographic distance (km) between populations measured using FOSSIL [50].

### Demographic inference in *G*. *maculatus*

We constructed genealogical relationships in *G*. *maculatus* using Maximum Parsimony Networks in Hapview (http://www.cibiv.at). To estimate the pattern of demographic history in the species, we compared the distribution of pairwise differences between haplotypes (mismatch distribution) for each locality, main area, and for the whole data set, to the expected distribution under the sudden expansion growth model of Rogers and Harpending [51]. This analysis is a popular method since the amount of nucleotide differences between haplotypes depends on the length of time since they diverged. Alternatively, we reconstructed past population dynamics through time using a Bayesian Skyline Plot method implemented in BEAST v. 1.7 [52]. This method is fundamentally based on coalescent theory, which quantifies the relationship between a genealogy of the sequences and the demographic history of a population. In contrast to the previous method, it does not assume any demographic model. For comparison purposes, three substitution models (strict clock, uncorrelated lognormal and uncorrelated relaxed clock) were computed for the main areas here analyzed and compared statistically using a Bayes factor test [53] with TRACER v. 1.5 (http://beast.bio.ed.ac.uk/Tracer). The uncorrelaed lognormal model was the best fit for D-loop data in *G*. *maculatus*. We conducted three independent Bayesian MCMC runs using the GTR + I + G model, previously estimated using MrModeltest v. 2.3 (http://www.abc-se/~nylander), and a specific population level mutational rate estimated for *G*. *maculatus* by Salinas [54]. Two independent runs per area (Intermediate Area and Magellanic Province) were made for 50 x 10^6^ generations (sampled every 1000 step), discarding a 10% of the trees as burn-in. The convergence of runs was confirmed with Tracer ensuring a minimum of 1000 effective sampling for each statistics (ESS). The results of the multiple runs were combined using LogCombiner [52]. The median and corresponding credibility intervals of the Bayesian skylines plots were depicted with Tracer.

### Gene flow and connectivity

We compared different models of gene flow between the main areas of interest (Intermediate Area and Magellanic) with the software MIGRATE v.3.5 [55], to test for different scenarios. For this, we defined four candidate models constraining the presence of directionality of gene flow between the main areas. The first model allowed bidirectional gene flow between the Intermediate Area and the Magellanic Province (full island model). Models 2 and 3 were defined by asymmetric patterns of gene flow from the Intermediate Area to the Magellanic Province, and from the Magellanic Province to the Intermediate Area, respectively. Finally, model 4 assumed both main areas as the same panmictic population. All the analyses were run using a GTR + I + G substitution model and transition-transversion ratio of 2.9818 as previously estimated by jModeltest v2 [56]. The specific substitution rate for the selected marker in the species was set to constant, as suggested by the manual. Analysis consisted of one long chain with 500.000 recorded parameter steps, a sampling interval of 100 and a burn-in of 100.000 (10%), running multiple replicates (10 independent chains). A heated scheme (1.00, 1.50, 3.00, and 1000000.0) was used to calculate the marginal likelihoods for model comparisons. Comparisons among the different models were done using a Bayes Factors calculated by substracting the highest value of the log marginal likelihoods (lmL; Bezier curve approximation) from the lmL values of each models. Finally, the associated probability of each model in relation to others was estimated following Kaas and Rafery [57].

We estimated different demographic indices and evaluated two asymmetric isolation-with-migration models between main areas of interest (Intermediate Area and the Magellanic Province) using IMa2 software [58,59]: i) from the Intermediate Area to Patagonia (m _N > S_) and from Patagonia to the Intermediate Area (m _S > N_). We carried out separately several preliminary runs in the M mode (Markov Chain Monte Carlo; MCMC mode) of the software to set the best set of priors that ensure mixing and convergence. Uniform priors were used to estimate effective population size (**Θ**_1_, **Θ**_2_, and ancestral **Θ**_a_, **Θ** = 3000) and splitting time (t = 300), whereas an exponential prior (mean = 1) for gene flow (m) was adopted. We performed 80 x 10^6^ MCMC steps sampling every 100 generations, with a burn-in of 8 x 10^6^. D-loop sequences were assumed to mutate under the HKY mutation model following the author guidelines [59]. Once convergence was achieved under the M-mode, we used the same simulated genealogies under the L-Mode (Load Tree mode) to estimate the log maximum-likelihood and credibility intervals (95% under HPD) estimates for migration parameters using a Likelihood Ratio Test. Under the L-Mode we compared our data with a null model of no migration [58,59]. Finally, estimated parameters were re-scaled into years (t/μ) and effective rate of migration ((**Θ**_x_/ m_x_)/2) (rate at which genes come into population, per generation) using an overall substitution rate per year (m = 1.1 x 10^−4^) estimated on previous studies [54].

## Results

**Fig 2:**
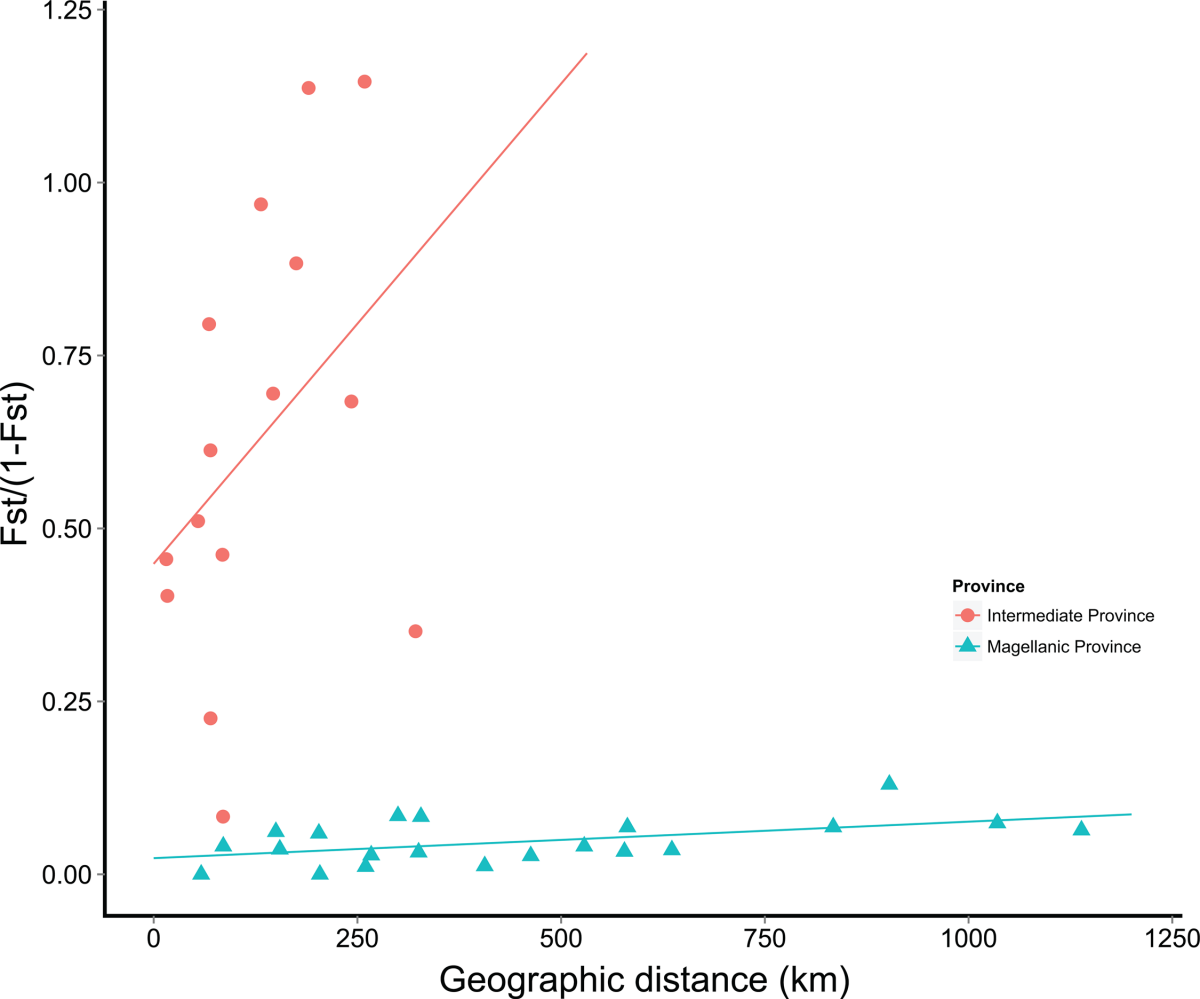
Relationship between linearized genetic differentiation (N_ST_) and geographic distances (Km) along the Intermediate Area and the Magellanic Province. Red circles and blue triangles represent pairwise values in the Intermediate Area and the Magellanic Province, respectively.

The whole D-loop data set in *G*. *maculatus* included 353 individuals and consisted in 926 nucleotide positions. As expected working with a non-coding region, several insertion and deletions were detected and were not considered in further analyses. Sequences were A-T rich (57.1%) compared to G-C content (42.9%). Levels of genetic diversity in the species were extremely high with 230 polymorphic characters (24.83%); and 156 of them (67.82%) were parsimoniously informative. In spite of the high level of genetic polymorphosm recorded in the species, D-loop sequences were not saturated. Haplotype diversity (*H*) ranged between 0.942 (Lingue River) and 1.000 (Moncul River, Queule River, Maullín River, Yelcho River, and María Eugenia Bay; Table 1). The average number of nucleotide differences (*П*) and nucleotide diversity (*π*) were very high in most of the localities and ranged between 30.55/0.033 (Chaihuín River) and 10.74/0.011 (Williams Channel). Levels of genetic diversity of *G*. *maculatus* were higher in Intermediate Area localities than in Magellanic ones, particular for *П* and *π* (Table 1). In fact, permutation test analyses (100,000 iterations) detected highly significant values for *П* (P = 0.00066) and *π* (P = 0.00063) in Intermediate Area populations compared to those from the Magellanic Province. Similarly, pairwise N_ST_ comparisons detected a high degree of genetic structure between Intermediate Area and Magellanic Province populations (Table 2). High levels of genetic structure, along a narrow geographical range (< 250 km), were detected among Intermediate Area localities. On contrast, low levels of genetic differentiation were recorded along the Magellanic Province (Table 2). Similarly, global S_nn_ analyses in the species (S_nn_ = 0.45) indicate moderate levels of phylogeographic signal in the whole data set. Nevertheless, S_nn_ statistic recognized a higher degree of phylogeographic signal along the Intermediate Area localities (S_nn_ = 0.63) than in Magellanic Province ones (S_nn_ = 0.43). General pattern of genetic structure registered in *G*. *maculatus* was supported by AMOVA analyses detecting 7 groups with a maximal difference accounting for 37.98% of the total variance, and only 2.01% was due to within-group variation among localities (Table 3). AMOVA analysis recognized each one the Intermediate locality as a separate population while all the localities along the Magellanic Province were recognized as a single unit. A moderate and marginally correlation between genetic and geographic distances was detected among Intermediate Area populations (r = 0.398; P = 0.087) and the Magellanic Province ones (r = 0.45; P = 0.08) (Fig 2).

**Table 1:** Diversity índices and neutrality tests in *Galaxias maculatus* along it distribution in the Chilean coast.

Locality	*n*	*K*	*H*	*S*	*П*	*П*	*π*	Tajima´s D	Fu´s FS	M. D.
Moncul River	24	24	1.000	85	20.20	0.022	-0.81	-10.16***	M
Queule River	23	23	1.000	85	22.93	0.025	-0.64	-8.55***	M
Lingue River	24	18	0.942	78	23.23	0.025	-0.010	-3.53*	M
Valdivia River	28	24	0.989	68	24.79	0.027	1.03	-3.76*	M
Chaihuín River	41	31	0.974	106	24.94	0.027	-0.56	-3.90*	M
Maullín River	27	27	1.000	112	30.55	0.033	-0.28	-9.12*	M
**Intermediate Area**	**167**	**145**	**0.993**	**180**	**32.61**	**0.035**	**-0.45**	**-33.37** ***	**M**
Yelcho River	22	22	1.000	72	15.84	0.017	-1.19	-10.52***	M
Concoto Island	26	24	0.994	54	10.88	0.011	-1.09	-12.27***	M
Williams Channel	26	20	0.978	42	10.74	0.011	-0.33	-5.24***	M
María Eugenia Bay	23	23	1.000	63	15.33	0.016	-0.66	-11.66***	M
Tortel	27	26	0.997	71	15.88	0.017	-1.01	-11.95***	M
Pascua River	45	43	0.998	75	13.24	0.014	-1.28	-32.40***	M
Strait of Magellan	17	16	0.993	51	15.52	0.016	-0.33	-4.32**	M
**Magellanic Province**	**186**	**170**	**0.998**	**142**	**14.11**	**0.015**	**-1.73**	**-261.20** ***	**M**
**Total**	**353**	**310**	**0.998**	**218**	**25.03**	**0.027**	**-1.27**	**-33.94** ***	**M**

*n* = number of analyzed individuals; *k* = number of haplotypes; *S* = polymorphic sites; *H* = haplotype diversity; *П =* average number of pairwise differences; *π =* nucleotide diversity. M.D. Mismatch Distribution, M = Multimodal.

*p<0.05

**p<0.01

*** p<0.001.

**Table 2:** Mean general pairwise values of differentiation (Ф_ST_) between *Galaxias maculatus* populations.

Locality	1	2	3	4	5	6	7	8	9	10	11	12	13
1	***												
2	**0.44**	***											
3	**0.31**	**0.28**	***										
4	**0.49**	**0.38**	**0.33**	***									
5	**0.41**	**0.12**	**0.18**	**0.31**	***								
6	**0.26**	**0.53**	**0.40**	**0.53**	**0.46**	***							
7	**0.60**	**0.11**	**0.42**	**0.46**	**0.12**	**0.66**	***						
8	**0.64**	**0.11**	**0.46**	**0.50**	**0.12**	**0.71**	0.05	***					
9	**0.64**	**0.14**	**0.46**	**0.50**	**0.11**	**0.70**	**0.07**	0.03	***				
10	**0.60**	**0.05**	**0.41**	**0.45**	**0.07**	**0.67**	**0.07**	0.05	0.03	***			
11	**0.60**	**0.06**	**0.42**	**0.45**	**0.08**	**0.66**	0.03	0.01	0.02	0.00	***		
12	**0.64**	**0.09**	**0.47**	**0.51**	**0.12**	**0.68**	0.06	0.02	0.03	0.01	0.00	***	
13	**0.59**	**0.09**	**0.39**	**0.45**	**0.09**	**0.66**	0.06	0.06	**0.11**	0.06	0.03	0.03	***

Where: 1) Moncul River; 2) Queule River; 3) Lingue River; 4) Valdivia River; 5) Chaihuín River; 6) Maullín River; 7) Yelcho River; 8) Concoto Island; 9) Williams Channel; 10) María Eugenia Bay; 11) Tortel; 12) Pascua River; 13) Strait of Magellan. Statistically significant comparisons (after Bonferroni correction) and 100,000 iterations are marked in bold.

**Table 3:** Analysis of Molecular Variance (AMOVA) depicting the percentage of variation explained among groups (Moncul River, Queule River, Lingue River, Valdivia River, Chaihuín River, Maullín River, and Magellanic Province populations), among populations within groups, and within populations. Where F_SC_ represents differntiation within populations among groups while F_CT_ represents differentiation among groups (*** p<0.001, ** p<0.01).

Source of variation		d.f.	Sum of squares	Variance components	Percentage of variation
Among groups		6	1571.864	6.14210 Va	36.47
Among populations within groups		6	88.683	0.18003 Vb	1.10
Within populations		340	3424.433	10.07186 Vc	61.44
Total		352	5084.980	16.39400	
Fixation Index					
F_SC_	0.01756***				
F_CT_	0.37466***				

**Fig 3:**
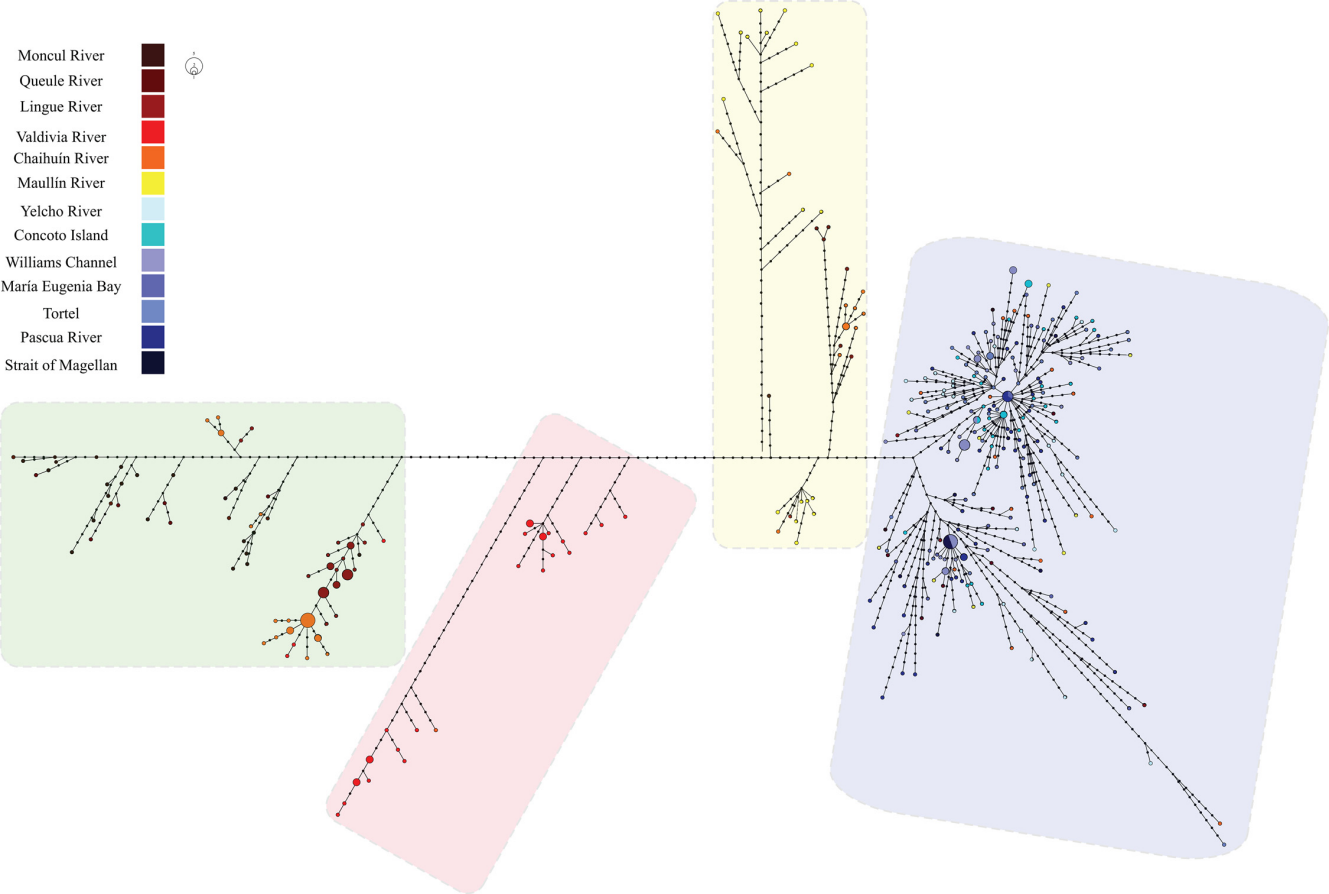
Maximum parsimony haplotype network including 353 individuals of *Galaxias maculatus* mtDNA D-loop sequences. Each haplotype is represented by a colored circle the locality where was collected. The size of the circles are proportional to its frequency in the whole sampling effort. Colored areas mark the different recognized haplogroups where Green (Hap 01), Red (Hap 02), Yellow (Hap 03), and blue (Hap 04).

**Fig 4:**
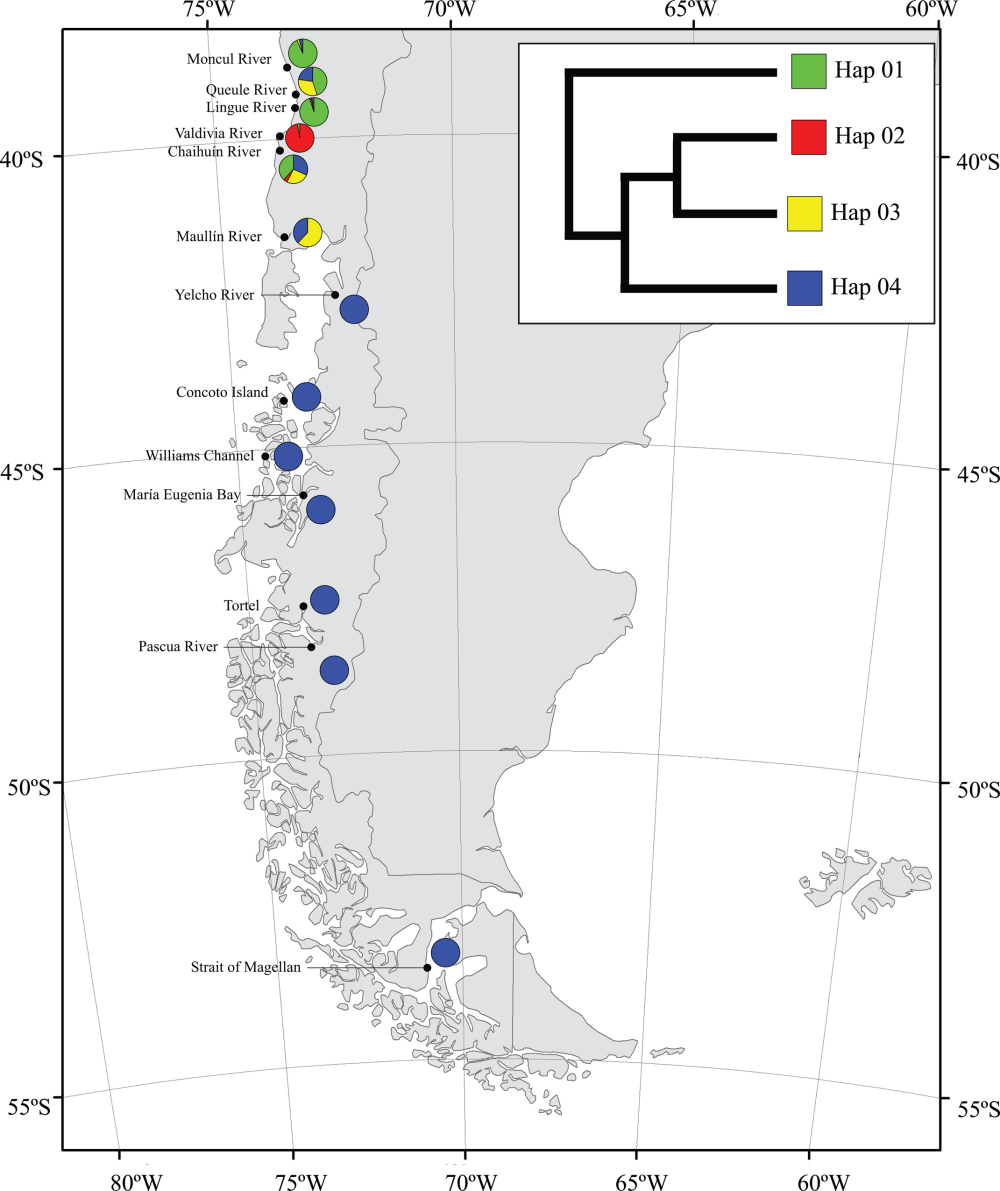
Distribution of the recognized haplogroups in *G*. *maculatus* along the sampling localities.

Maximum Parsimony haplotype network in *G*. *maculatus* recovered a total of 321 haplotypes and a very expanded genealogy (Fig 3). A total of 298 haplotypes (92.83%) were unique and only 23 haplotypes were present in more than two individuals. As previously recognized through mean standard diversity indices, Intermediate Area localities showed a more expanded genealogies than Magellanic Province ones. Four main groups of haplotypes (haplogroups) were detected in *G*. *maculatus*. Two of them (Hap 01 and Hap 03; Fig 4) include individuals collected at Intermediate Area localities from Moncul to Maullín. Haplogroup 02 was endemic at Valdivia River and was also present in a single specimen from Lingue River. The fourth haplogroup (Hap 04) includes individuals from Magellanic Province localities, from Yelcho River to the Strait of Magellan. Interestingly, all the diversity recorded in the Magellanic province fell within the Patagonian haplogroup 04 recorded by Zemlak et al [22]. Some individuals from Intermediate Area localities, particularly from Maullín, Chaihuín and Queule rivers, were also found within the southern haplogroup (Fig 4).

Tajima’s D and Fu’s F_S_ neutrality tests showed similar trends at both sectors and for the whole D-loop data set in *G*. *maculatus*. Tajima’s D test was negative but not statistically significant for each locality, area, and for the whole D-loop data set. In contrast, Fu’s F_S_ test was negative and statistically significant for all localities, area, and for the whole data set (Table 1). Furthermore, analyses of pairwise differences in *G*. *maculatus* recovered multimodal distribution of values with multiple peaks in each of the analyzed localities, area, and for the whole data set (Table 1).

**Fig 5:**
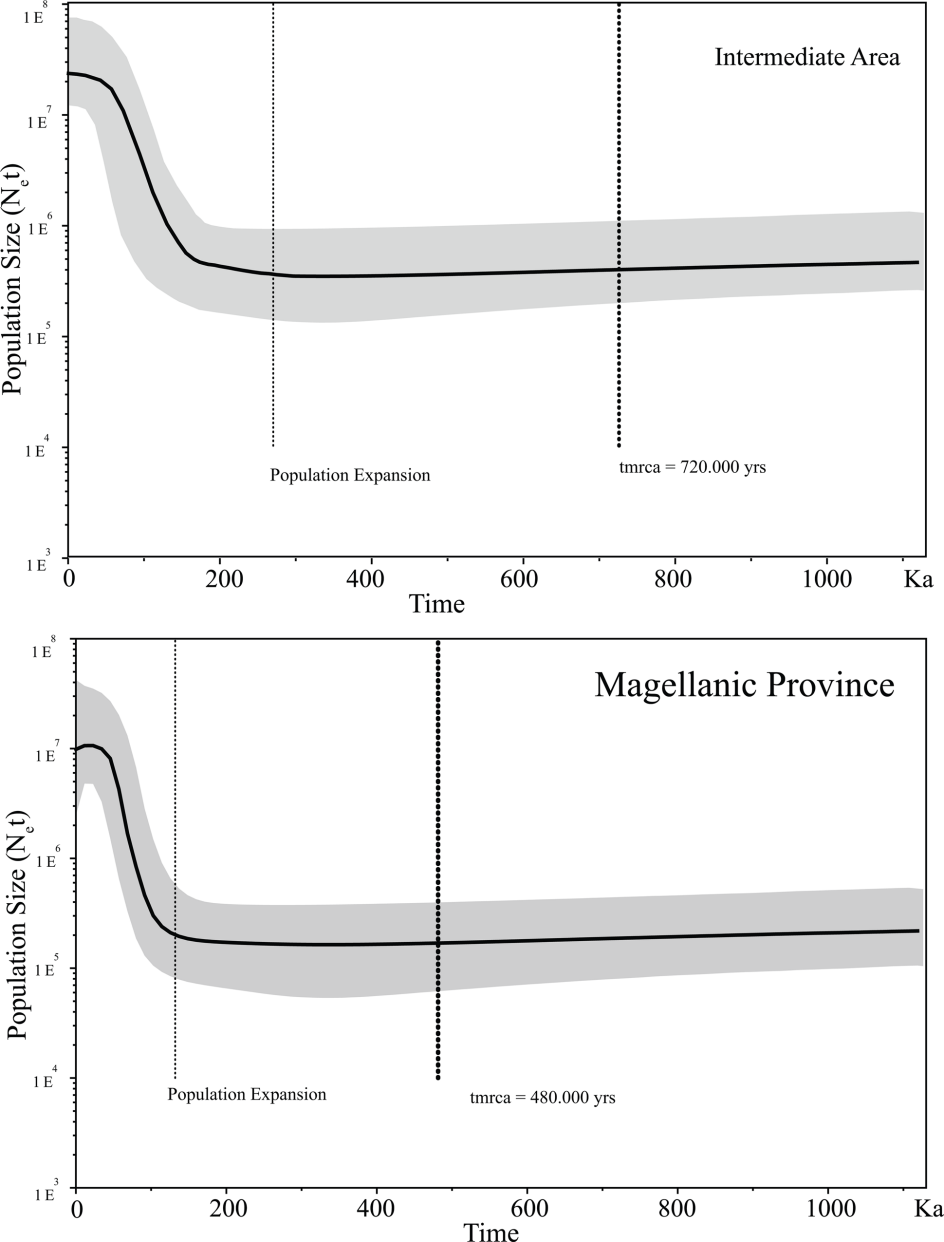
Historical demographic trends of the effective population sizes (N_e_) constructed using a Bayesian skyline plot approach based on D-loop haplotypes of *Galaxias maculatus* along its distribution in the Chilean coast (Intermediate Area and Magellanic Province). The y-axis is the product of effective population size (N_e_) and generation length in a log scale while the x-axis is the time 10^3^ before present. The median estimate (solid line) and 95% highest probabilities density (HPD; grey areas) are shown. The thick dashed line represent the time of the most common ancestor (trmca) and the thin dashed line represents time for population expansion.

Bayesian Skyline plot analyses recognized differences in the times of the most recent common ancestor (tmrca) and population expansions between the Intermediate Area and the Magellanic Province. Based on these analyses, the trmca of the Intermediate Area occurred ∼ 72 Ka while the tmcra for the Magellanic Province occurred ∼ 48 Ka (Fig 5). Similarly, the onset of the population expansion in the Intermediate Area occurred ∼ 32 Ka against the ∼ 18 Ka estimated for the Magellanic Province.

Gene flow analyses using MIGRATE and different migration models detected evidence of asymmetrical gene flow from the Magellanic Province to the Intermediate Area. Among the tested models the one from the Magellanic Province to the Intermediate Area received the highest probability (Table 4). Similarly, coalescent approach of isolation-with-migration implemented in IMA2 detected overall low levels of gene flow between the Intermediante Area and Magellanic Areas localities. However, the model predicted a most likely pattern of asymmetric migration with greater migration from the Magellanic Province to the Intermediate Area (Table 5).

**Table 4:** Log marginal likelihoods (lmL) and log Bayes factor (LBF) comparisons for different migration models between the Intermediate Area and the Magellanic Province in *G*. *maculatus*. Where IA = Intermediate Area; MP = Magellanic Province.

Model	Bezier lmL	LBF	Model prob	Model rank
1. full migration	-8199.540	-55.779	<0.001	3
2. IA to MP	-8180.607	-17.920	<0.001	2
**3. MP to IA**	**-8171.654**	**0**	**1**	**1**
4. panmixia	-8397.543	-451.779	<0.001	4

**Table 5:** Estimation of isolation-with-migration model implemented in IMa2 including; migration rates (m) in each direction (m_N->S_, m_S->N_), estimated splitting time (τ) between provinces (in years ago), and effective population size of both provinces (Θ_N_, Θ_S_) and ancestral population size (Θ_A_) converted in demographic units (individuals).

	m _N->S_	m _S->N_	τ (YA)	Θ_N_	Θ_S_	Θ_A_
Hight Point	0.0005ns	0.034ns	28,347	1,636,744	637,824	2,992,261
95% HPD	0.0005–0.1285	0.0025–0.1285	22,948–36,447	995,545–2,987,760	43,534–907,802	2,048,461–4,513,139

For each parameter, the most probable value (high point) and the 95% highest posterior density (95% HPD) of the marginal posterior probabilities are shown. Ns = non significant for the LTR for migration rates estimation. The convertion of scale in the τ and Θ parameter was carried on using a specific substitution rate of 12% per million year estimated by Salinas [52].

## Discussion

Molecular-based genetic studies have become pivotal to further understand and unravel how Quaternary glacial cycles affected the distribution and demography of populations, species, and communities [3,4,60–65]. Evidence of postglacial recolonization of the Magellanic Province has been recorded in freshwater [22,25,66] and marine fishes [67,68], lizards [28], amphibians [27], mammals [9,10,69], marine invertebrates [6–8], and plants [70,71]. Traditional genetic models of glacial refugia and recolonization routes have been proposed to describe the response of populations, species, and communities to climatic changes [62–65]. The Expansion-Contraction Model propose that species would have become restricted to glacial refugia at lower latitude outside the influence of glacial ice advances during cooling periods. After this, they expanded their distribution towards previously glaciated areas at higher latitudes following the deglaciation process [62–64]. Therefore, unglaciated and refugial areas are expected to harbor higher levels of genetic diversity than peripheral, geologically altered, or newly founded regions. On contrast, formerly glaciated areas should exhibit evidence of recent postglacial demographic expansions. According to Maggs et al. [63], formerly glaciated areas should also exhibit small number of haplotypes dominating disproportionately large areas as a consequence of postglacial recolonization processes. Simultaneously, recolonized areas should exhibit low divergence among haplotypes and lower levels of genetic differentiation than refugial ones.

### Main patterns of genetic diversity and structure in *G*. *maculatus*

Even if coastal and Andean South American samples of *G*. *maculatus* have been previously included in phylogeographic and demographic studies [22,39], here we present the first population-level study in the species along two biogeographical provinces that were differentially affected during the glacial cycles of the Quaternary. In contrast to previous studies that incorporated mainly river and lake specimens, our study focuses on estuarine and coastal populations. As previously determined in the species [22,39,41], *G*. *maculatus* harbors extremely high levels of genetic diversity along its distribution in the Chilean Pacific coast. In fact, our results further corroborate previous molecular studies in the species [22,41,72], pointing towards an extreme within-species genetic diversity among coastal and estuarine populations. In this study, out of 353 individuals we recognized 321 different haplotypes. Similarly, Zemlak [22] recorded a total of 273 different haplotypes, out of 299 individuals, while Waters et al. [72] recognized 139 different haplotypes out of 144 individuals from New Zealand. In spite of the high levels of genetic polymorphism recorded in this study, we also detected marked and significant differences in the average number of nucleotide differences and nucleotide diversity between main biogeographical areas here included. Therefore, higher levels of genetic diversity along formerly unglaciated areas (Intermediate Area) than in those located within the limits of the Patagonian Ice Sheet (Magellanic Province) detected in the species, supports the first prediction of the Expansion-Contraction model. Main differences recorded in terms of genetic diversity between the main biogeographical areas here included were also supported by population dynamics reconstructions showing a more recent population expansion in the Magellanic Province than in the Intermediate Area. Therefore, our results support the hypothesis that the Intermediate Area represents an older region and less affected by glacial advances and retreats than the Magellanic Province. Together with main patterns of genetic diversity recorded in the species we also found contrasting patterns of genetic structure between the main biogeographic areas here analyzed. Each one of the analyzed rivers along the Intermediate Area, expanding less than 250 km, represented a different genetic unit that generally coincided with the defined haplogroups of Zemlak et al. [22] at the same localities. Magellanic populations of *G*. *maculatus*, expanding for more than 1000 km, showed low levels and even absence of genetic differentiation. Again, major differences in terms of the genetic structure between formerly glaciated and unglaciated areas are adjusted to the predictions of the Expansion-Contraction model. Nevertheless, high levels of genetic diversity, measured as *П* and *π*, among Magellanic localities and the absence of dominant broadly distributed haplotypes in this area do not fit with the expectations of recent postglacial expansion from refugial areas at lower latitudes. Moreover, the presence of a markedly differentiated and highly diverse haplogroup (Hap04) in this Province does not support the idea of a recent postglacial recolonization. Such results contrast to those observed in other South American freshwater [24,25,73–76] and marine fishes [67,68]. In fact, Alò et al. [75] recorded low levels of genetic diversity and a clear genetic continuity between Intermediate Area and Magellanic province populations of *Aplochiton* (*A*. *zebra*, *A*. *taenius* and *A*. *marinus*). Most of these studies have demonstrated the important role of the last glacial cycle over the demography of Magellanic fishes with the presence of typical star-like haplotype genealogies as a consequence of recent colonization processes associated to strong founder effects. In this context, population genetic patterns in Patagonian terrestrial plants and vertebrates indicate that different processes and directional range shifts have generated a mosaic of phylogeographical patterns that are far more complex than a simple north to south described one [71]. In the particular case of *G*. *maculatus*, Zemlak et al. [22,39] proposed that the main patterns of genetic diversity and structure in the species suggest a low amount of influence of the last glacial cycle. In fact, levels of genetic diversity along the Magellanic Province likely reflect the existence of marine *in situ* or periglacial refugia during cooling periods of the Quaternary.

### Patterns of genetic structure in *G*. *maculatus* along the analyzed areas

Low genetic differentiation along the Magellanic Province, a complex geographic and oceanographic area, may be a response of particular characteristics of the species. The southern fjords and channels ecosystems along the Pacific coast of Patagonia are considered as one of the largest estuarine ecosystem in the planet [77,78]. Subantarctic waters masses are mixed with freshwater ones from abundant precipitation, river flow, and glacial meltwater [79]. A low salinity surface layer generates a highly stable water column within the fjords whereas a well-mixed water column occurs in the gulfs and open channels. In this context, epipelagic *G*. *maculatus* larvae have been reported in ichthyological samples along the Magellanic Province [78]. Considering these findings and the pattern of genetic structure recorded in the species it seems like the Magellanic Province constitutes a continuous habitat for *G*. *maculatus*. In contrast, genetic structure along the Intermediate Area suggests low levels of connectivity between river basins. In this context, open sea areas between the coastal rivers here analyzed seem to be preventing the genetic continuity of the species along the Intermediate Area. Such results are quite surprising considering the high dispersal potential described for New Zealand coastal populations of *G*. *maculatus* [80]. In this context, mechanisms associated to larval retention or physiological limitation associated to salt tolerance may explain the complex pattern of genetic structure along the Intermediate Area. Otherwise, philopatric behavior could also generate small-scale genetic structure within the Intermediate Area localities, despite the high dispersal potential of the species [34].

Major differences in terms of genetic diversity and structure recorded in *G*. *maculatus* agree with the 42°S biogeographic break described along the Chilean coast resulting from different oceanographic, geologic, and climatic processes [38,77]. Along the Chilean coast, this break represents a boundary for major environmental changes in terms of topography, currents, and water salinity that represent a major transition for marine [38] and freshwater species [81]. Different biogeographic scenarios altered the species assemblages across this limit and likely generated distinct patterns of genetic diversity [82]. First, this break coincides with the separation of the West Wind Drift into two main oceanic currents along the Pacific coast [39]. Intermediate Area localities are washed by the northward flow of the Humboldt Current System [83] while the Magellanic Province is located within the influence of the Cape Horn Current, the southern branch of the West Wind Drift. In this context, the presence along Intermediate Area localities of haplotypes belonging to the Magellanic diversity (Hap 04) points towards asymmetric northward gene flow that could be associated to a latitudinal displacement of the West Wind Drift during glacial periods [84,85]. A concordance between phylogeographic and biogeographic patterns has been recorded in many areas of the globe indicating that the forces determining species distributions are also related to the spatial patterns of population genetic structure [86–90]. Therefore, local adaptation processes and distinctive characteristics of main biogeographic areas along the Chilean coast in terms of Quaternary glacial histories and oceanographic regimes are responsible for the general pattern of genetic structure recorded in *G*. *maculatus*.
